# Protein ontology on the semantic web for knowledge discovery

**DOI:** 10.1038/s41597-020-00679-9

**Published:** 2020-10-12

**Authors:** Chuming Chen, Hongzhan Huang, Karen E. Ross, Julie E. Cowart, Cecilia N. Arighi, Cathy H. Wu, Darren A. Natale

**Affiliations:** 1 Department of Computer and Information Sciences, University of Delaware, Newark, Delaware 19716 USA; 2 Department of Biochemistry and Molecular & Cellular Biology, Protein Information Resource, Georgetown University Medical Center, Washington, DC 20007 USA

**Keywords:** Data integration, Data mining

## Abstract

The Protein Ontology (PRO) provides an ontological representation of protein-related entities, ranging from protein families to proteoforms to complexes. Protein Ontology Linked Open Data (LOD) exposes, shares, and connects knowledge about protein-related entities on the Semantic Web using Resource Description Framework (RDF), thus enabling integration with other Linked Open Data for biological knowledge discovery. For example, proteins (or variants thereof) can be retrieved on the basis of specific disease associations. As a community resource, we strive to follow the Findability, Accessibility, Interoperability, and Reusability (FAIR) principles, disseminate regular updates of our data, support multiple methods for accessing, querying and downloading data in various formats, and provide documentation both for scientists and programmers. PRO Linked Open Data can be browsed via faceted browser interface and queried using SPARQL via YASGUI. RDF data dumps are also available for download. Additionally, we developed RESTful APIs to support programmatic data access. We also provide W3C HCLS specification compliant metadata description for our data. The PRO Linked Open Data is available at https://lod.proconsortium.org/.

## Introduction

As an evolving extension to the current hypertext document web, Linked Open Data (LOD) is a new paradigm where data are published and interconnected on the web using open standards such as Uniform Resource Identifiers (URIs), Hypertext Transfer Protocol (HTTP), Resource Description Framework (RDF), Web Ontology Language (OWL) and SPARQL Protocol and RDF Query Language (SPARQL). This enables data from heterogeneous sources to be shared, integrated and queried in a web of data. Tim Berners-Lee in his web architecture note introduced a set of best practices for publishing and interlinking structured data on the web, also well known as the Linked Data principles^1^: (1) Use URIs as names of things; (2) Use HTTP URIs, so that people can look up those names; (3) When someone looks up a URI, provide useful information using the standards (RDF, SPARQL etc.); and (4) Include links to other URIs, so that they can discover more things.

Bio2RDF^2^ is an open source project that uses Semantic Web technologies to build a large network of Linked Open Data for Life Sciences from a diverse set of heterogeneously formatted sources obtained from multiple data providers. It uses federated SPARQL queries to facilitate continuous integration of life sciences data from resources such as Mouse Genome Informatics (MGI)^3^, Saccharomyces Genome Database (SGD)^4^, Rat Genome Database (RGD)^5^, NCBI’s Gene resources (NCBIGene)^6^, HUGO Gene Nomenclature Committee (HGNC)^7^, and Nematode Information Resource (WormBase)^8^. The European Bioinformatics Institute (EBI) provides an RDF platform^9^ that facilitates answering complex research questions through queries/exploration of integrated resources, including UniProtKB^10^, Reactome^11^, Ensembl^12^, and Gene Ontology^13^.

As formal and explicit specifications of domains of interest, ontologies consist of terms representing precisely defined entities and the relationships between them. Ontologies are increasingly being used to define the basic terms and relations in biological domains, often as the foundation for search, integration and exchange of biological data. The Protein Ontology (PRO)^14^ provides an ontological representation of protein-related entities, notably, including those of organism-neutral nature^15^. In addition to the ontology itself, PRO includes annotations and cross-reference information, and can be used to facilitate the discoverability and aggregation of data related to protein entities in the context of protein functions, pathways and drug targets. The Protein Ontology has been used by the research community for applications including named entity recognition/tagging^16–18^, entity definition (for example, of cell types^19^), and as an import for protein-related terms in other ontologies^20,21^. PRO Linked Open Data and SPARQL endpoint have been used to assist in orthology mapping^22^ and to study kinase post-translational modifications and cancer-associated mutations^23^. The PRO website has garnered more than 20 million hits in the past year. PRO Linked Open Data and SPARQL endpoint alone garnered 321,000 hits. PRO is ranked among the top 5 endpoints by YummyData SPARQL endpoint monitor^24^.

In this paper we introduce the PRO RDF data models and the metadata description of PRO Linked Open Data. We provide faceted browser, SPARQL endpoint with graphical user interface (GUI), and downloadable RDF files. We demonstrate federated SPARQL queries to answer questions for biological knowledge discovery using multiple connected resources.

## Results

**Fig. 1:**
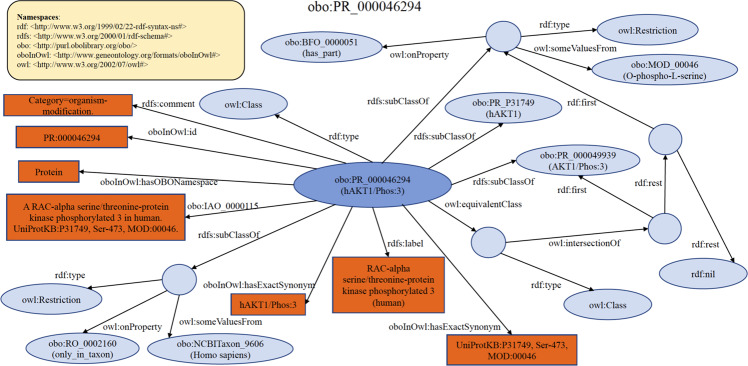
A PRO RDF data model (PR:000046294). Ellipse and circle shapes are RDF nodes. Rectangle shapes are RDF literals. Directed edges are RDF properties. Circle shapes represent anonymous classes or blank nodes. ‘AKT1’, used here for brevity, is the gene for ‘RAC-alpha serine/threonine-protein kinase’.

Each PRO term represents a distinct class of organism-neutral or organism-specific protein entities (e.g., modified forms, orthologous isoforms, and protein complexes). To help readers understand PRO RDF data models, we present an exemplar PRO term in each category using RDF Graph, RDF/XML, and Turtle formats. Links for these are provided in the relevant section, however an example RDF Graph of one PRO term (PR:000046294, RAC-alpha serine/threonine-protein kinase phosphorylated 3 (human)) is also shown in Fig. 1 and listed as Turtle format below.

@prefix owl:<http://www.w3.org/2002/07/owl#>.

@prefix rdfs:<http://www.w3.org/2000/01/rdf-schema#>.

@prefix xsd:<http://www.w3.org/2001/XMLSchema#>.

@prefix obo:<http://purl.obolibrary.org/obo/>.

@prefix oboInOwl:<http://www.geneontology.org/formats/oboInOwl#>.

obo:PR_000046294 a                owl:Class;

rdfs:comment   “Category = organism-modification.”;

rdfs:label                       “RAC-alpha serine/threonine-protein kinase phosphorylated 3 (human)”;

rdfs:subClassOf      obo:PR_000049939, obo:PR_P31749;

rdfs:subClassOf      [a                   owl:Restriction;

owl:onProperty              obo:RO_0002160;

owl:someValuesFrom obo:NCBITaxon_9606             

];

rdfs:subClassOf   [a              owl:Restriction;

                     owl:onProperty        obo:BFO_0000051;

                     owl:someValuesFrom obo:MOD_00046

                  ];

obo:IAO_0000115          “A RAC-alpha serine/threonine-protein kinase phosphorylated 3 in human. UniProtKB:P31749, Ser-473, MOD:00046.”;

oboInOwl:hasDbXref                    “Reactome:R-HSA-377265”;

oboInOwl:hasExactSynonym “UniProtKB:P31749, Ser-473, MOD:00046”, “hAKT1/Phos:3”;

oboInOwl:hasOBONamespace “protein”;

oboInOwl:id      “PR:000046294”;

owl:equivalentClass             [a                           owl:Class;

                        owl:intersectionOf (obo:PR_000049939

                                                                     [a                                   owl:Restriction;

                             owl:onProperty        obo:RO_0002160;

                             owl:someValuesFrom obo:NCBITaxon_9606])].

                                                      ]

                                                    )

               ].

obo:RO_0002160          rdfs:label        “only_in_taxon”.

obo:NCBITaxon_9606          rdfs:label                          “Homo sapiens”.

obo:PR_000049939                 oboInOwl:hasExactSynonym “AKT1/Phos:3”.

obo:PR_P31749                           oboInOwl:hasExactSynonym   “hAKT1”.

obo:BFO_0000051           rdfs:label                             “has_part”.

obo:MOD_00046             rdfs:label                            “O-phospho-L-serine”.

### PRO uniform resource identifiers (URIs)

PRO follows the rule of reusing existing identifiers whenever feasible, e.g., http://purl.obolibrary.org/obo/GO_0032991 (Gene ontology), http://purl.obolibrary.org/obo/RO_0002160” (Relations Ontology), etc. The format of PRO identifier is “PR_xxxxxxxxx”, e.g., http://purl.obolibrary.org/obo/PR_000000001. However, for UniProtKB related entries incorporated into PRO organism-gene or organism-sequence categories, we use the UniProtKB accession number and prefix it with “PR_” as its corresponding PRO identifier. e.g., http://purl.obolibrary.org/obo/PR_P00015.

### Types of PRO terms

Protein Ontology terms are organized into general categories, often with organism-specific versions. Below we describe these categories. Example terms for each category are given in Table 1. Whenever possible, PRO attempts to make connections between orthologs. Extending from the concept of orthology between genes, we have introduced the concept of orthologous proteoforms. For example, ortho-isoforms are isoforms that are believed to have arisen prior to speciation and divergence of the primary sequence. That is, ortho-isoforms were true alternative isoforms (as defined above) in a common ancestor and are, quite likely, functionally equivalent. Ortho-modified forms are modified versions of ortho-isoforms where the modification(s) occur on equivalent residues.

**Table 1:** Example PRO terms in each category.

Category		Term	Name	Link to Example
Family	organism-neutral	PR:000000027	smad protein	https://lod.proconsortium.org/rdf.html#category_family
	organism-specific	PR:000044507	14-3-3 protein (human)	https://lod.proconsortium.org/rdf.html#category_org_family
Gene	organism-neutral	PR:000000364	smad2	https://lod.proconsortium.org/rdf.html#category_gene
	organism-specific	PR:000022736	fumarate hydratase class II	https://lod.proconsortium.org/rdf.html#category_org_gene
SeqGroup	organism-neutral	PR:000050216	receptor-type tyrosine-protein phosphatase C isoform CD45R	https://lod.proconsortium.org/rdf.html#category_seqgroup
	organism-specific	PR:Q9ULB1	neurexin-1-alpha (human)	https://lod.proconsortium.org/rdf.html#category_org_seqgroup
Sequence	organism-neutral	PR:000000048	TGF-beta receptor type-2 isoform RII-1	https://lod.proconsortium.org/rdf.html#category_sequence
	organism-specific	PR:Q68FF6-1	ARF GTPase-activating protein GIT1 isoform 1 (mouse)	https://lod.proconsortium.org/rdf.html#category_org_sequence
Modification	organism-neutral	PR:000049939	RAC-alpha serine/threonine-protein kinase phosphorylated 3	https://lod.proconsortium.org/rdf.html#category_modification
	organism-specific	PR:000046294	RAC-alpha serine/threonine-protein kinase phosphorylated 3 (human)	https://lod.proconsortium.org/rdf.html#category_org_modification
Complex	organism-neutral	PR:000027291	phosphorylase kinase complex PHKL	https://lod.proconsortium.org/rdf.html#category_complex
	organism-specific	PR:000036137	lipopolysaccharide receptor complex 3; endosome membrane (human)	https://lod.proconsortium.org/rdf.html#category_org_complex

#### Category = family

Each PRO term at the family level refers to protein products of a distinct gene family arising from a common ancestor. The leaf-most nodes at this level are usually families comprising paralogous sets of gene products (of a single or multiple organisms). For example, smad2 and smad3 both encode proteins that are TGF receptor-regulated while smad1, smad5, and smad9 are all BMP receptor-regulated. Thus, “TGF-beta receptor-regulated smad protein” and “BMP receptor-regulated smad protein” are terms denoting distinct families. Note that this level collectively refers to any such grouping at any level of similarity. For example, the two families indicated above can merge into a “receptor-regulated smad protein” class and further merge (with the protein products of smad4, smad6, and smad7) into the “smad protein” class.

#### Category = gene

Each PRO term at the gene level refers to the protein products of a distinct gene in a reference organism and the orthologs thereof. For example, “smad2” and “smad3” are two different genes, and therefore have two different PRO entries at the gene level of distinction. The protein products of what is recognized as smad2 in humans and what is recognized as smad2 in mouse fall under the single gene-level term “smad2”. Thus, a single term at the gene-level distinction collects the protein products of (usually 1-to-1) orthologs for that gene. Organism-specific versions are (typically) defined logically as the intersection of the parent (gene-level) and the organism (taxonomic) terms. If a resource provides gene information, PRO will indicate this using the *has_gene_template* relation.

#### Category = seqgroup

Each PRO term at the seqgroup level refers to proteins encoded by the same gene that are distinguished from siblings based on differences in shared portions of encoding mRNAs. That is, all members of a given seqgroup are encoded by mRNAs that have a common subset of sequence features, either as a common subset of exons or as a common subset of sequence variations. Examples include proteins encoded by the PTPRC (CD45) gene, where each member of the CD45R subtype (CD45RA, CD45RAB, CD45RAC) minimally contains exon 4 (aka ‘A’) even while each member of a given subtype has other exons that make them distinct, and histocompatibility genes such as HLA-A, where each member of a given subtype (HLA-A*24, HLA-A*68, etc) shares a common set of variations even while each member of a given subtype has other variations that make them distinct.

#### Category = sequence

Each PRO term at the sequence level refers to the protein products that arise from different alleles of a given gene (sequence variants), from splice variants of a given RNA, or from alternative initiation and ribosomal frameshifting during translation. One can think of this as a mature mRNA-level distinction. For example, smad2 encodes both a long splice form and a short splice form. The protein products of each isoform are separate PRO terms. If there is clear knowledge on the equivalency of isoforms (that is, they are “ortho-isoforms”)^14^, then the equivalent terms from different organisms are defined as children of the sequence level terms. For example, ARF GTPase-activating protein GIT1 isoform 1 from mouse (PR:Q68FF6-1), ARF GTPase-activating protein GIT1 isoform 1 from rat (PR:Q9Z272-1), and ARF GTPase-activating protein GIT1 isoform 3 from human (PR:Q9Y2X7-3) are ortho-isoforms that are children of the sequence level term PR:000044155. If the equivalency of isoforms is not well-established, the organism-sequence term is defined as a child of the organism-gene level term.

#### Category = modification

Each PRO term at the modification level refers to the protein products derived from a single mRNA species that differ because of some change (or lack thereof) that occurs after the initiation of translation (co- and post-translational). This includes sequence differences due to cleavage and chemical changes to one or more amino acid residues. In general, PRO does not provide time or space information, i.e., the order in which modifications occur or the location in which they are found unless such are required to properly define the term. PRO only represents the final modified objects. If there is clear knowledge on the equivalency of the modified forms from different organisms, then the equivalent organism-modification terms are defined as children of the modification level terms. For example, RAC-alpha serine/threonine-protein kinase phosphorylated 3 from human (PR:000046294) and mouse (PR:000049940) are ortho-modified forms that are phosphorylated on the position equivalent to Ser-473 of the human protein. In the PRO hierarchy, they are children of the modification level term PR:000049939. If the equivalency of the modified forms is not well-established, the organism-modification term is defined as a child of the appropriate organism-gene term.

#### Category = complex

Each PRO term at the complex level^25^ ultimately traces to the general term “protein-containing complex” found in GO (GO:0032991), defined as “A stable assembly of two or more macromolecules, i.e. proteins, nucleic acids, carbohydrates or lipids, in which at least one component is a protein and the constituent parts function together”. Indeed, complex-level terms in PRO are imported from GO whenever possible. However, because GO complexes are (predominantly) defined with respect to function, PRO will create complex terms when a component-specific definition is desired. When doing so, PRO represents complexes explicitly–that is, by subunit composition–defining each member of the complex at the level of its isoform, variant, or modified form, whenever possible. Most complex-related terms defined by PRO are organism-specific. Note that a protein in its monomeric state, linked non-covalently to a small chemical, is not considered a protein complex by the above definition, but it can be defined as a subclass of the protein term using the CHEBI ID for the small chemical and the relation “non-covalently_bound_to”.

### Dataset description

A high-quality consistent metadata description is essential to the successful discovery, exchange, and query of a Linked Dataset. The Protein Ontology Linked Open Data is accompanied with metadata description using Vocabulary of Interlinked Datasets (VoID), the Provenance vocabulary (PROV) and Dublin Core vocabulary, which are compliant with the W3C HCLS specification^26^. The metadata for Protein Ontology Linked Open Data is described at three levels: (1) summary level, which provides a description of a dataset that is independent of a specific version or format; (2) version level, which captures version-specific characteristics of a dataset; and (3) distribution level, which captures metadata about a specific form and version of a dataset.

Linkset is a way of identifying the content that links instances in one dataset with instances in another dataset. A separate linkset is created for each link predicate relating a particular pair of datasets. A linkset is a subset of the dataset which publishes it. The linkset itself is of type void:Linkset and provides the same metadata as a RDF distribution. The statistics relevant for a linkset are the number of triples it contains and are reported using the void:triples property. The full VoID description of PRO Linked Open Data is at https://lod.proconsortium.org/releases/latest/void.ttl.

### Federated SPARQL query

Federated SPARQL query is a powerful method that allows the simultaneous search of multiple data resources and aggregates the results from multiple SPARQL endpoints. PRO RDF data can be combined with other RDF data, such as UniProt^10^ RDF data (at https://sparql.uniprot.org/) and DisGeNET^27^ RDF data (at http://rdf.disgenet.org/sparql/) as demonstrated below to generate new biological insights that are not possible using one individual resource.

#### Sample federated SPARQL query 1

**Fig. 2:**
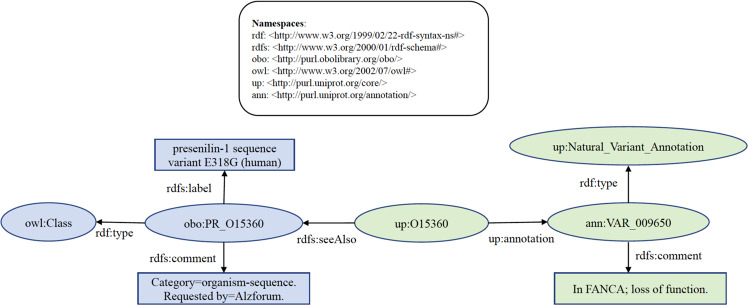
Knowledge graph of exemplary query result of federated SPARQL query 1 (Get all human genes in PRO whose UniProtKB counterpart has variants with loss of function implicated in disease). Ellipse shapes are RDF nodes. Rectangle shapes are RDF literals. Directed edges are RDF properties.

To answer the question, “Which human proteins are potentially involved in disease via loss of function”, the query is designed to get all human proteins in PRO whose UniProtKB counterpart has variants with loss of function implicated in disease. The partial query result is shown in Fig. 2 as a knowledge graph.

Query 1:

PREFIX obo:<http://www.geneontology.org/formats/oboInOwl#>

PREFIX up:<http://purl.uniprot.org/core/>

PREFIX ud:<http://purl.uniprot.org/database/>

PREFIX up:<http://purl.uniprot.org/core/>

PREFIX taxon:<http://purl.uniprot.org/taxonomy/>

PREFIX rdf:<http://www.w3.org/1999/02/22-rdf-syntax-ns#>

PREFIX rdfs:<http://www.w3.org/2000/01/rdf-schema#>

PREFIX faldo:<http://biohackathon.org/resource/faldo#>

SELECT DISTINCT?

PRO_term

(STR(?_PRO_label) AS?PRO_label)

(STRAFTER(STRBEFORE(STR(?_PRO_category), “.”), “ = “) AS?PRO_category)

?Protein

?Variant

(STR(?_Text) AS?Description)

WHERE

{

SERVICE<http://sparql.uniprot.org/sparql>

{

?Protein rdfs:seeAlso?PRO_term.?

PRO_term

up:database ud:PRO;

a up:Resource.?Protein a up:Protein.?Protein up:organism taxon:9606.?Protein up:annotation?Variant.?Variant a up:Natural_Variant_Annotation.?Variant rdfs:comment?_Text.

FILTER (CONTAINS(?_Text, ‘loss of function’))

}

?PRO_term rdfs:label?_PRO_label.

?PRO_term rdfs:comment?_PRO_category.

}

#### Sample federated SPARQL query 2

**Fig. 3:**
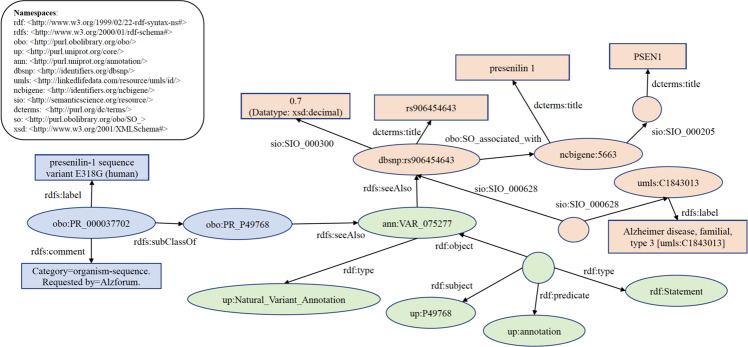
Knowledge graph of exemplary query result of federated SPARQL query 2 (Find variants in UniProt or DisGeNET for AlzForum PRO terms). Ellipse and circle shapes are RDF nodes. Rectangle shapes are RDF literals. Directed edges are RDF properties. Circle shapes represent anonymous classes or blank nodes.

To answer the question, “What disease(s) are associated with AlzForum-derived sequence variants described in PRO”, we construct a query to find variants in UniProt or DisGeNET for AlzForum (https://www.alzforum.org/) PRO terms. The partial query result is shown in Fig. 3 as a knowledge graph.

Query 2:

PREFIX obo:<http://purl.obolibrary.org/obo/>

PREFIX up:<http://purl.uniprot.org/core/>

PREFIX ud:<http://purl.uniprot.org/database/>

PREFIX taxon:<http://purl.uniprot.org/taxonomy/>

PREFIX rdf:<http://www.w3.org/1999/02/22-rdf-syntax-ns#>

PREFIX rdfs:<http://www.w3.org/2000/01/rdf-schema#>

PREFIX sio:<http://semanticscience.org/resource/>

PREFIX ncit:<http://ncicb.nci.nih.gov/xml/owl/EVS/Thesaurus.owl#>

PREFIX dcterms:<http://purl.org/dc/terms/>

PREFIX so:<http://purl.obolibrary.org/obo/SO_>

PREFIX oboInOwl:<http://www.geneontology.org/formats/oboInOwl#>

PREFIX skos:<http://www.w3.org/2004/02/skos/core#>

SELECT DISTINCT

?PRO

(?PRO_Label AS?Name)

(STRAFTER(STRBEFORE(STR(?PRO_Category), “.”), “ = “) AS?Category)

(STRBEFORE(STRAFTER(STR(?PRO_Category), “.”), “.”) AS?Comment)

(?protein AS?UniProt)

(?uniprotvar AS?UniProt_Var)

(?variant AS?DisGeNET_Var)

(STR(?variantTitle) AS?RS_ID)

(STR(?vscore) AS?VDAScore)

(?disease AS?Disease)

(STR(?diseaselabel) AS?DiseaseName)

(?gene AS?Gene)

(STR(?gSymbol) AS?GeneSymbol)

(STR(?geneTitle) AS?GeneName)

WHERE {

{

SELECT?PRO?Parent?PRO_Label?PRO_Category?uniprotvar

WHERE {

VALUES?PRO {obo:PR_000037702 obo:PR_000037753}

?PRO rdfs:subClassOf?Parent.

?Parent rdfs:seeAlso?uniprotvar.

?PRO rdfs:label?PRO_Label.

?PRO rdfs:comment?PRO_Category.

FILTER(CONTAINS(LCASE(?PRO_Category), “by = alzforum”))

FILTER(CONTAINS(LCASE(STR(?uniprotvar)), “annotation”))

}

}

SERVICE<http://sparql.uniprot.org/sparql>

{

?uniprotvar

a up:Natural_Variant_Annotation;

rdfs:seeAlso?_rs;

skos:related?up_disease.

?up_disease

a up:Disease;

rdfs:comment?up_disease_comment.

FILTER(CONTAINS(LCASE(?up_disease_comment), “alzheimer”))

[]

rdf:object?uniprotvar;

rdf:predicate up:annotation;

rdf:subject?protein;

a rdf:Statement.

BIND(IRI(REPLACE(STR(?_rs), “purl.uniprot.org”, “identifiers.org”)) AS?variant)

}

SERVICE<http://rdf.disgenet.org/sparql/>

{

OPTIONAL {

?vda sio:SIO_000628?variant,?disease;

sio:SIO_000216?vscoreIRI.?vscoreIRI sio:SIO_000300?vscore.?variant dcterms:title?variantTitle.?disease rdfs:label?diseaselabel.?disease dcterms:title?diseaseTitle.

FILTER(CONTAINS(LCASE(?diseaseTitle), “alzheimer”))

OPTIONAL {?variant so:associated_with?gene.?gene a ncit:C16612.?gene dcterms:title?geneTitle.?gene sio:SIO_000205?symbolUri.?symbolUri dcterms:title?gSymbol.

}

}

}

} ORDER BY DESC(?vscore)

## Discussion

The guiding principles of FAIR data aim to facilitate the discovery, integration, and analysis of relevant datasets by humans and machines by making them Findable (F), Accessible (A), Interoperable (I), and Reusable (R)^28^. As a community resource, we strive to follow FAIR principles, disseminate regular updates of our data, support multiple methods for accessing, querying and downloading data in various formats, and provide documentation both for scientists and programmers. The formal FAIRness assessment^29,30^ results for Protein Ontology Linked Open Data are presented in Table 2. According to the results, PRO follows the FAIR principles quite well. Moreover, according to the YummyData SPARQL endpoint monitor, PRO (https://yummydata.org/endpoint/129) ranks among the top 5 endpoints by the Umaka Score^24^ calculated based on six criteria: Availability, Freshness, Operation, Usefulness, Validity and Performance.

**Table 2:** The formal FAIRness assessment results for Protein Ontology Linked Open Dataset.

Metric	Requirement	Resource	Resource data/content
F1A	IRI for a registered identifier scheme for your resource’s IRI	PURL schema	PURL schema
		http://purlz.org	http://purlz.org
F1B	IRI to a document describing the persistency policy for the identifier of this data	http://purlz.org	http://purlz.org
			https://lod.proconsortium.org/rdf.html#uri
F2	IRI for machine-readable metadata for the resource	https://lod.proconsortium.org/releases/latest/void.ttl	https://lod.proconsortium.org/PR_000025934
	IRI to file format for this metadata	https://www.w3.org/TR/void/	https://www.w3.org/TR/void/
F3	Is the resource identifier specified in the metadata?	Yes	Yes
F4	URL to a search engine indexing your resource	https://www.google.com	https://www.google.com
	Search query/terms	“Protein Ontology” - > First hit	“PR_000025934” - > First hit
A1.1	URL to the description of the protocol	HTTP	HTTP
		https://en.wikipedia.org/wiki/Hypertext_Transfer_Protocol	https://ecciki/Hypertext_Transfer_Protocol
	Is the protocol open?	Yes	Yes
	Is the protocol (royalty) free?	Yes	Yes
A1.2	Is authorization required to access the content of your resource?	No	No
A2	URL to metadata longevity plan	https://lod.proconsortium.org/release.html	Provided at the dataset level
I1	URL to a specification language	RDFS and OWL ontology	RDFS and OWL ontology
		https://www.w3.org/TR/rdf-schema/	https://www.w3.org/TR/rdf-schema/
		https://www.w3.org/TR/owl2-overview/	https://www.w3.org/TR/owl2-overview/
I2	Maximum 3 IRIs for vocabularies used within the (meta)data	http://purl.org/dc/terms/	dcterms:title “PRO Linked Open Data”@en
		http://www.w3.org/ns/dcat#	dcat:keyword “Protein Ontology”^^xsd:string, “Linked Open Data”^^xsd:string
		http://www.w3.org/2004/02/skos/core#	void:linkPredicate skos:closeMatch
I3	URL to a LinkSet (https://www.w3.org/TR/void/) for the resource	https://lod.proconsortium.org/releases/latest/void.ttl	Provided at the dataset level
R1.1	URL to license/terms of use for the resource	https://creativecommons.org/licenses/by/4.0/	
R1.2	Maximum 3 IRIs used to describe the provenance of the resource	http://purl.org/dc/terms/	dcterms:accrualPeriodicity freq:quarterly
		http://xmlns.com/foaf/0.1/	dcterms:publisher [foaf:page]
		http://purl.org/pav/	pav:hasCurrentVersion:prolod59_0
	Maximum 3 IRIs used to describe domain information	http://purl.obolibrary.org/obo/pr#	oboInOwl:hasSynonymType pr:PRO-short-label
		http://purl.obolibrary.org/obo/	obo:PR_Q7TMZ5
		http://www.geneontology.org/formats/oboInOwl#	oboInOwl:hasSynonymType pr:PRO-short-label
R1.3	IRI that represents certification from a recognized authority	http://yummydata.org	Provided at the dataset level

Nonetheless, there are still some improvements to be made, and these will be addressed in the future work. For example, we plan to improve PRO entry pages to support content negotiation with structured data, such as JSON-LD to provide explicit clues about the meaning of the PRO term to search engines.

PRO has a user-friendly web interface that supports search, browse and retrieval of PRO terms and related information at https://proconsortium.org/. The PRO website also provides a query interface to the SPARQL endpoint, and new functionality using Linked Open Data is being developed. Web statistics show an increasing proportion of accesses to the SPARQL query interface over text searches, indicating the trends towards using the SPARQL server and programmatic access.

## Methods

Data imported into PRO comes largely from well-known curated sources (for example, UniProtKB and Reactome) that are updated with each release through a combination of manual and automated means. Special attention is given to terms for which positional information is cited. Upon import, and regularly thereafter, such positions are verified against the current protein sequence (for example, to ensure that a named amino acid is at the indicated position on the sequence). Data integrity and consistency of ontology is checked prior to each release using the ELK reasoner^31^, a very fast reasoner that supports the EL subset of OWL 2 as part of the ROBOT tool^32^.

Users can query PRO Linked Open Data using SPARQL at https://sparql.proconsortium.org/virtuoso/sparql, which is powered by OpenLink Virtuoso server community edition (http://vos.openlinksw.com/owiki/wiki/VOS) (version 07.20.3217) with the faceted browser, SPARQL 1.1 query federation and Cross-Origin Resource Sharing (CORS) enabled to support a range of complex and federated queries that merge data from other SPARQL endpoints.

### Faceted browser

**Fig. 4:**
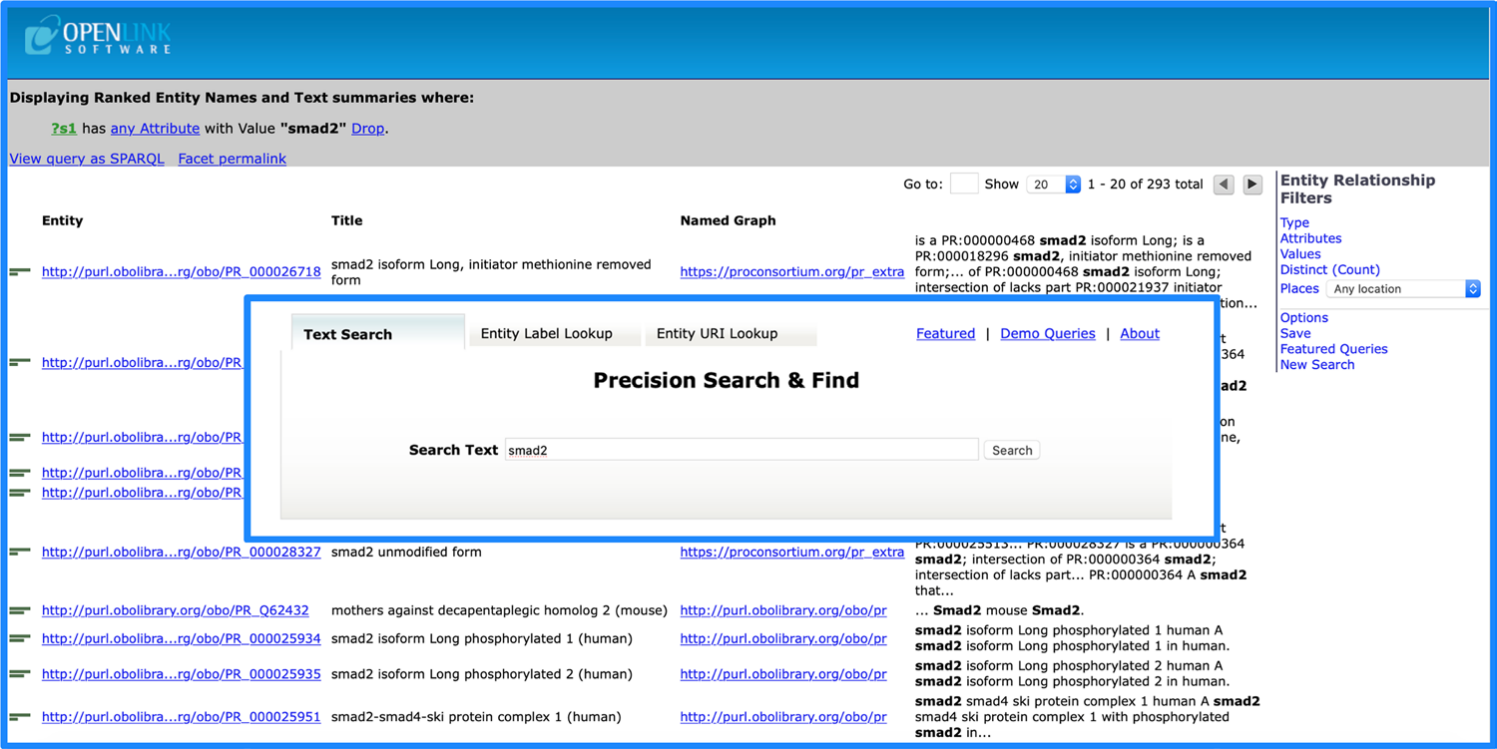
Virtuoso faceted browser query interface and result table view.

Protein Ontology Linked Open Dataset can be accessed via Virtuoso Faceted Browser (Fig. 4), a general-purpose RDF data query facility for data exploration by faceted browsing over entity relationship types (i.e. relations).

### SPARQL GUI

**Fig. 5:**
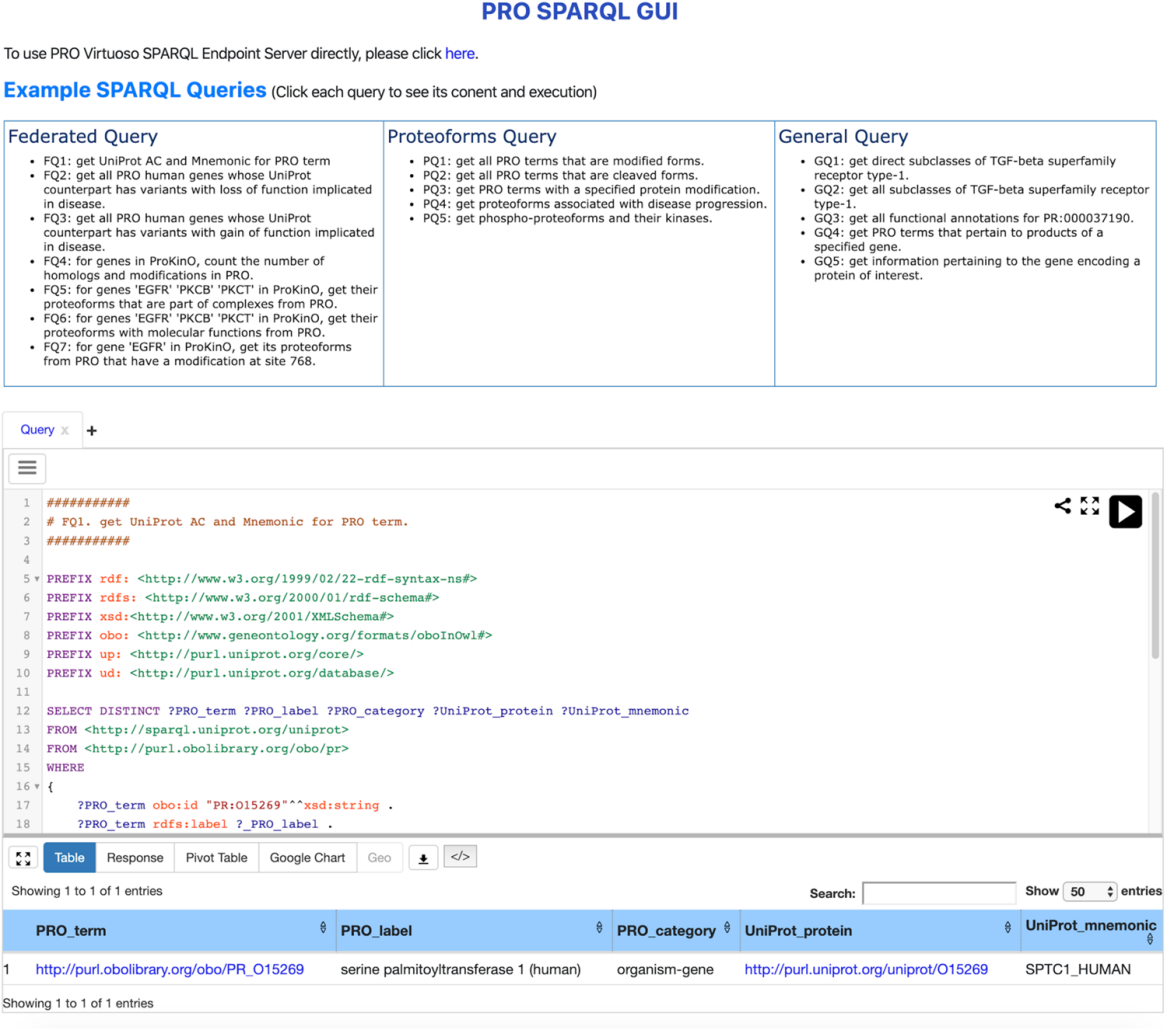
PRO LOD SPARQL GUI. It provides users with a portal to query Protein Ontology Linked Open Data using the SPARQL 1.1 standards as well as a comprehensive set of example queries.

Protein Ontology Linked Open Data can be accessed via YASGUI (Yet Another Sparql GUI), a web application to query any SPARQL endpoint. YASGUI provides various advanced features for creating, sharing, and visualizing SPARQL queries and their results. We also provided a comprehensive set of example SPARQL queries (Fig. 5).

### RESTful APIs

**Fig. 6:**
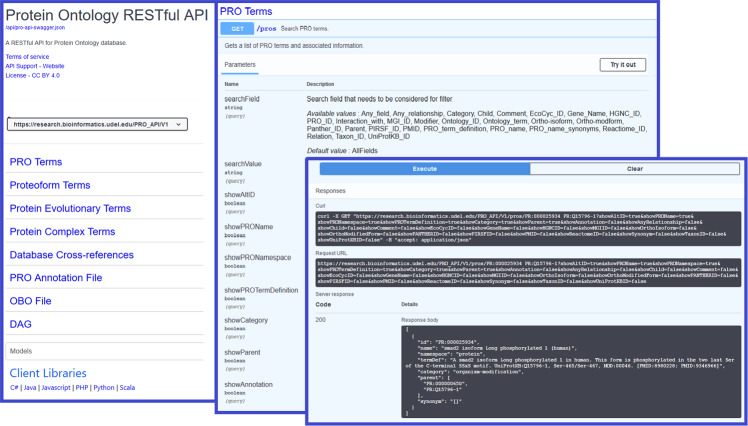
API documentation for Protein Ontology Linked Open Data. The Swagger™ API generates an interactive webpage where users can ‘try out’ the service with real queries. Results are returned in the ‘Response Body’ in the user selected response format (JSON illustrated) or XML.

As a full query language, SPARQL can be difficult for some people to learn. We therefore developed RESTful APIs (Fig. 6) for programmatic access to Protein Ontology Linked Open Data for data integration or analysis. The API specification was designed using the Swagger™ Editor based on OpenAPI Specification 3. Swagger UI was used to visualize and interact with the API’s resources automatically generated from API specifications. The PRO APIs include 8 API operation groups and 34 access paths as shown in Table 3 and are implemented using the Django-REST framework.

**Table 3:** Currently supported Protein Ontology RESTful API endpoints.

API Operation Group	API Access Path*	Description
PRO Terms	/pros	Search PRO terms.
	/pros/{proIds}	Return PRO terms by IDs.
Proteoform Terms	/proforms/modification	Returns a list of modified protein forms.
	/proforms/modification/phosphorylated	Returns a list of phosphorylated protein forms.
	/proforms/modification/methylated	Returns a list of methylated protein forms.
	/proforms/modification/acetylated	Returns a list of acetylated protein forms.
	/proforms/modification/ubiquitinated	Returns a list of ubiquitinated protein forms.
	/proforms/modification/glycosylated	Returns a list of glycosylated protein forms.
	/proforms/orthoisoform	Returns a list of ortho-isoform protein forms.
	/proforms/orthomodform	Returns a list of ortho-modform protein forms.
	/proforms/sequence	Returns a list of sequence level protein forms.
	/proforms/organism-sequence	Returns a list of organism-sequence level protein forms.
Protein Evolutionary Terms	/proevos/family	Returns a list of family level protein terms.
	/proevos/gene	Returns a list of gene level protein terms.
	/proevos/organism-gene	Returns a list of organism-gene level protein terms.
Protein Complex Terms	/procomps/organism	Returns a list of organism specific protein complex terms.
	/procomps	Returns a list of organism non-specific protein complex terms.
Database Cross-references	/dbxrefs/EcoCyc_ID	Returns a list of PRO terms with EcoCyC ID as cross-reference.
	/dbxrefs/HGNC_ID	Returns a list of PRO terms with HGNC ID as cross-reference.
	/dbxrefs/MGI_ID	Returns a list of PRO terms with MGI ID as cross-reference.
	/dbxrefs/Ontology_ID	Returns a list of PRO terms with Ontology ID as cross-reference.
	/dbxrefs/PANTHER_ID	Returns a list of PRO terms with PANTHER ID as cross-reference.
	/dbxrefs/PIRSF_ID	Returns a list of PRO terms with PIRSF ID as cross-reference.
	/dbxrefs/PMID	Returns a list of PRO terms with PMID as cross-reference.
	/dbxrefs/Reactome_ID	Returns a list of PRO terms with Reactome ID as cross-reference.
	/dbxrefs/NCBITaxon_ID	Returns a list of PRO terms with NCBI Taxon ID as cross-reference.
	/dbxrefs/UniProtKB_ID	Returns a list of PRO terms with UniProtKB ID as cross-reference.
PRO Annotation File	/paf/{proIds}	Returns annotations for the given PRO ID(s).
OBO File	/obo/{proIds}	Returns PRO term in OBO format for the given PRO ID(s).
DAG	/dag/parent/{proIds}	Returns direct parent PRO terms by the given PRO ID(s).
	/dag/ancestor/{proIds}	Returns direct and indirect parent PRO terms by the given PRO ID(s).
	/dag/children/{proIds}	Returns direct children PRO terms by the given PRO ID(s).
	/dag/descendant/{proIds}	Returns direct and indirect children PRO terms by the given PRO ID(s).
	/dag/hierarchy/{proId}	Returns hierarchy of PRO terms by the given PRO ID.

*After “https://research.bioinformatics.udel.edu/PRO_API/V1”.

Users can use the API web interface to interactively customize the API requests. The API web pages also provide users with dynamically generated source code (client libraries) for common scripting and programming languages that provide examples with their query options and filters fully integrated that can be used as standalone scripts or programs or integrated into users’ large projects.

### RDF dumps

RDF dumps of Protein Ontology Linked Open Data are available for download in application/rdf + xml or text/turtle formats with corresponding RDF centric statistics, as shown in Table 4. Each dataset is loaded into a named graph in the Virtuoso triple store. A set of six RDF centric metrics are computed for each dataset that summarize their contents:

- total number of triples
- total number of distinct classes (the number of distinct resources occurring as objects of rdf:type)
- total number of entities
- total number of distinct subject nodes
- total number of distinct predicates
- total number of distinct object nodes

**Table 4:** RDF centric statistics for Protein Ontology Linked Open Dataset (release 61.0).

Dataset	Named Graph	Triples	Classes	Entities	Subjects	Predicates	Objects
pro	http://purl.obolibrary.org/obo/pr	11,858,7202	8	1,996,180	2,285,489	48	3,1298,222
paf	http://pir.georgetown.edu/pro/paf	91,407	4	8,313	19,733	22	31,301
pro-ensembl-closeMatch-linkset	https://lod.proconsortium.org/ensembl-closeMatch	48	0	0	24	2	24
pro-ensemblbacteria-closeMatch-linkset	https://lod.proconsortium.org/ensemblbacteria-closeMatch	1,758	0	0	493	2	879
pro-hgnc-closeMatch-linkset	https://lod.proconsortium.org/hgnc-closeMatch	36,542	0	0	18,268	2	18,271
pro-mgi-closeMatch-linkset	https://lod.proconsortium.org/mgi-closeMatch	31,024	0	0	15,509	2	15,512
pro-ncbigene-closeMatch-linkset	https://lod.proconsortium.org/ncbigene-closeMatch	5,310	0	0	1,693	2	2,655
pro-reactome-closeMatch-linkset	https://lod.proconsortium.org/reactome-closeMatch	29,042	0	0	10,169	2	14,521
pro-reactome-exactMatch-linkset	https://lod.proconsortium.org/reactome-exactMatch	43,998	0	0	11,220	3	14,666
pro-rgd-closeMatch-linkset	https://lod.proconsortium.org/rgd-closeMatch	14,690	0	0	7,343	2	7,345
pro-sgd-closeMatch-linkset	https://lod.proconsortium.org/sgd-closeMatch	2,522	0	0	1,261	2	1,261
pro-uniprotkb-closeMatch-linkset	https://lod.proconsortium.org/uniprotkb-closeMatch	274,308	0	0	54,960	2	133,016
pro-uniprotkb-exactMatch-linkset	https://lod.proconsortium.org/uniprotkb-exactMatch	527,418	0	0	175,793	3	169,975
pro-uniprotkbvar-closeMatch-linkset	https://lod.proconsortium.org/uniprotkbvar-closeMatch	938	0	0	41	2	469
pro-uniprotkbvar-exactMatch-linkset	https://lod.proconsortium.org/uniprotkbvar-exactMatch	1,407	0	0	469	3	469
pro-wormbase-closeMatch-linkset	https://lod.proconsortium.org/wormbase-closeMatch	3,570	0	0	1,785	2	1,785
void	https://sparql.proconsortium.org/.well-known/void	2,326	7	74	292	63	606

SPARQL queries used to calculate statistics can be found at https://code.google.com/archive/p/void-impl/wikis/SPARQLQueriesForStatistics.wiki.

The triples in “exactMatch-linkset” use “skos:exactMatch” as linkPredicate. The triples in “closeMatch-linkset” use “skos:closeMatch” as linkPredicate.

## Data Availability

RDF dumps can be downloaded at https://lod.proconsortium.org/release.html. Virtuoso faceted browser can be accessed at https://sparql.proconsortium.org/virtuoso/fct/. RESTful APIs can be accessed at https://lod.proconsortium.org/api.html. SPARQL GUI can be accessed at https://lod.proconsortium.org/yasgui.html. The formal FAIRness assessment results can be accessed at https://lod.proconsortium.org/fair.html.

## Code availability

The source code that implements https://lod.proconsortium.org/ website is available from GitHub at https://github.com/PROconsortium/PRoteinOntology/tree/master/pro_lod

The source code that implements PRO RESTful APIs is available from GitHub at https://github.com/PROconsortium/PRoteinOntology/tree/master/proapi
